# Evaluation of knowledge, experience and perceptions about medical emergencies amongst dental graduates (Interns) of Belgaum City, India

**DOI:** 10.4317/jced.50627

**Published:** 2012-02-01

**Authors:** Praveen S. Jodalli, Anil V. Ankola

**Affiliations:** 1 MDS.Senior lecturer, Department of Public health Dentistry, Yenepoya dental college & hospital, Mangalore; 2 Professor and Head, Department of Public health Dentistry, KLE Vishwanath Katti Institute of Dental sciences Belgaum, Karnataka

## Abstract

Introduction: Medical emergencies can occur frequently in dental setting. Effective management of an emergency situation in the dental office is ultimately the dentist’s responsibility. The lack of training and inability to cope with medical emergencies can lead to tragic consequences and sometimes legal action. For this reason, all health professionals including dentists must be well prepared to attend to medical emergencies. Providing basic life support [BLS] is dentist’s most important contribution until definitive treatment for a medical emergency can be given. Hence the study is aimed to assess the dental graduates (Interns) knowledge, skills and competency regarding dentistry-medical emergency interface. Method: Data were collected from 105 Interns of two dental colleges of Belgaum City using a structured questionnaire consisting of 7 item questionnaire (6 closed and 1 open ended). Results: Overall in all the years, syncope / faint was seen by 40.9% of the respondents, 37.1% with hypoglycemic attacks, allergic reactions by 17.1%, epileptic attacks by 7.6%, asthmatic attacks by 4.5% and angina 0.9%. The frequencies of the emergencies encountered were once or even more. Knowledge of the presence of drugs and equipments in the emergency drug kit and the confidence in regard to use them was low. Medical emergencies training were undertaken by 42% respondents, but in varying degrees. There was a desire for further medical emergencies training by majority of respondents. Conclusion: The study showed that syncope is the commonest medical emergency event. Dental graduates had a superficial knowledge of medical emergencies, drugs and equipments. Emphasis is placed on the need for more medical emergencies training to be offered, to increase knowledge and confidence of dental graduates (Interns) in the management of medical emergencies.

** Key words:**Medical emergencies, dental graduates, basic life support, emergency drugs.

## Introduction

Medical emergencies can be alarming to any clinician but these situations are less alarming if proper preparations are made. Medical emergencies occur in dental practice more frequently (1). Fortunately, serious medical emergencies in dental practice are not common but they are all the more alarming when they occur (2). A thorough patient history can draw the practitioner’s attention to potential medical emergencies that could occur (1). Changing demographics in the population, leading to increased longevity have led the people having medical conditions which predispose to a medical emergency or taking medication may influence their dental management and persons aged above 65 years or over are considered to be taking medication with a potential effect on dental care (3).

An increasing proportion of the population is medically at risk. According to the European resuscitation council, sudden cardiac arrest is a leading cause of death in Europe, affecting about 7, 00,000 individuals a year (4).

Thus, an effective management of an emergency situation in the dental office is ultimately the dentist’s responsibility. Although a number of studies have been carried out which sought to ascertain the emergency drugs and equipments, the lack of training and inability to cope with medical emergencies can lead to tragic consequences and sometimes legation action (5). For this reason, as all the health professionals, dentists must be well prepared to attend to and collaborate with the medical emergencies (6). Providing basic life support (BLS) is the dentists’ most important contribution until definitive treatment for a medical emergency can be provided (5).

Few studies have assessed how competent dentists consider themselves in managing medical emergencies, and very few studies to our knowledge have reported studies involving fresh dental graduates (7).

The aim of this study is to learn the experience of handling medical emergencies, their skills and competency and how well they felt are prepared to manage such events with appropriate use of drugs and equipments in a dental setting.

## Material and Methods

A cross sectional questionnaire approach was chosen to probe dental graduates’ (Interns) knowledge, experience and perceptions of medical emergency in the dental office. This research was conducted at KLE Vishwanath Katti Institute of Dental sciences and Maratha Mandal Dental College Belgaum, India during the academic year 2010.

A total of hundred and five (105) Bachelor of Dental Surgery graduates from the respective institutions undergoing the Internship program and who could look back over their experiences regarding the dental-medical emergency interface volunteered to participate, in response to an invitation issued in the college.

Respondents were told the study was completely confidential and encouraged to answer a 7 item pretested questionnaire (6 closed ended and 1 open ended) which was in a tick box format. Data collection procedures followed the standards of the KLE VK Institute of dental sciences Research and Ethical committee, Belgaum. All the graduates (Interns) signed a consent form.

The purpose of this study was to evaluate the dental graduates, knowledge, experience, and perceptions regard-ing medical emergencies in the dental practice. The questionnaire sought information on the frequency and type of medical emergencies encountered by the interns in the past 4 years.

● The knowledge, confidence of administering the essential drugs and equipments required to be in the emer-gency drug box (8).

● The amount of medical emergencies training undertaken by participants on the past 4 years and what percent of this was basic life support training (9).

● If participants felt that more training of general medical emergencies is required.

Completed questionnaire were collected personally and data were subjected to descriptive analysis using SPSS version 18.0 to identify the most frequently occurring medical emergencies.

## Results

Hundred and five (105) out of hundred and fifteen (115) dental graduates answered the questionnaire (91.3%). Sixty-one (61) of hundred and five (58.1%) had experienced an emergency situation during their graduation.

 Syncope / faint was seen by 40.9% of the respondents, 37.1% with hypoglycemic attacks, allergic reactions by 17.1%, epileptic attacks by 7.6%, asthmatic attacks by 4.5% and angina 0.9%. The frequencies of the emergen-cies encountered were once or even more.

( Table 1, Table 2) shows the percentage of dental graduates about the knowledge of identifying the recommended drugs, essential pieces of equipments in the emergency kit and the confidence of using them.

**Table 1:**
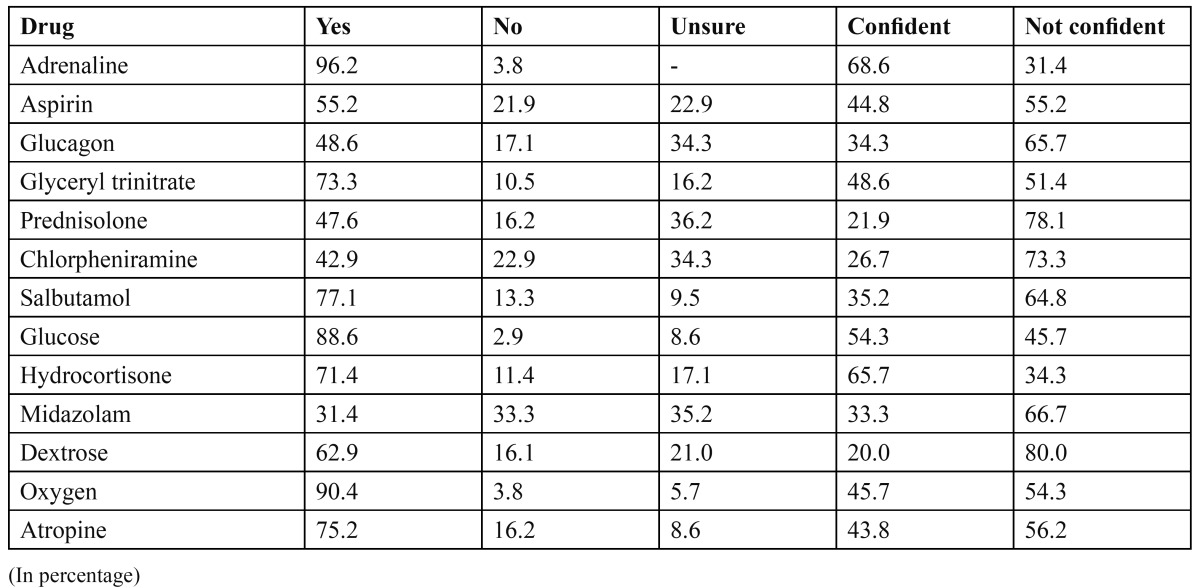
Essential drugs recommended being present in the drug kit and the level of confidence in using them.

**Table 2:**
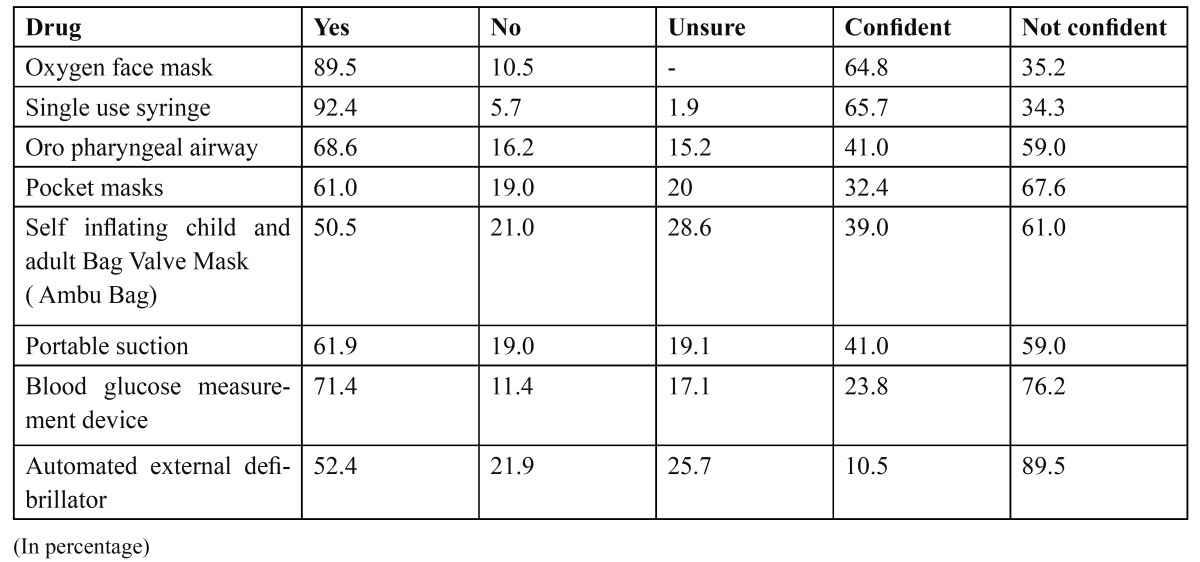
Essential equipments recommended being present in the kit and the level of confidence in using them.

Forty- five out of hundred and five (42.9%) had received no medical emergency training, and the remaining sixty (57.1%) had received a medical emergency and basic life support (BLS) training for less than 5 hours.

Hundred and two graduates (97.1%) felt the need for more medical emergencies training.

( Table 3) shows the competency in the areas of drug administration/other procedures.

**Table 3:**
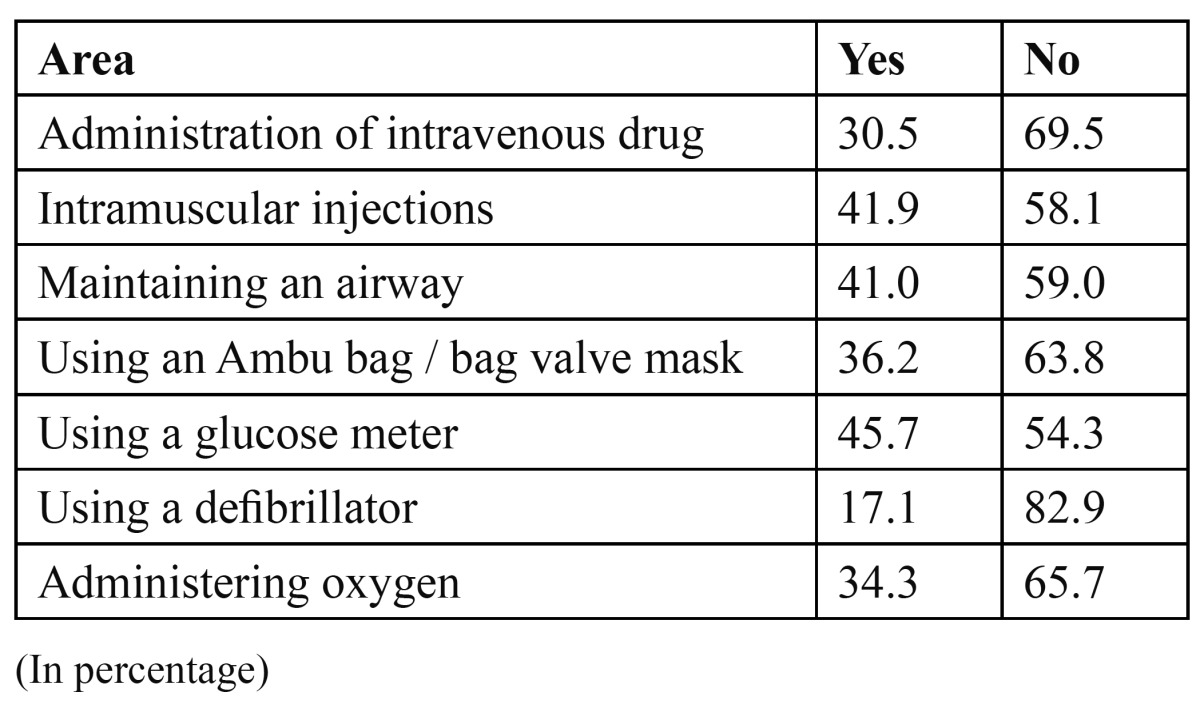
Do you feel competent in the following areas.

From an open ended question, we observed that the knowledge of the dental graduates (Interns) was at an acceptable level as majority of them expressed to terminate the treatment and activate the emergency services (EMS) when any emergency situation in the dental office occurs.

## Discussion

The results of this study confirmed that the dental graduates (Interns) are not capable of competently managing a medical emergency and perceived a need for more intensive education in medical emergencies, and they strongly desire to obtain this knowledge (5,10).

Several studies have assessed qualified dental surgeons on management of medical emergencies, (4,11,12,13) but to our knowledge there is no reported study involving fresh Dental graduates (Interns). So this cohort was targeted to probe and quantify the perceptions of medical emergency in the dental office.

Although there have been relatively few studies carried out regarding medical emergencies in dental hospitals, our study results show that the proportion of specific medical emergency events occurring were similar with those studies (9,11,14). The most commonly encountered emergencies seen by the Interns in both the colleges were syncope/faints, followed by asthmatic and hypoglycemic attacks. This would indicate that the training should be focused on dealing with these emergencies.

A higher frequency sixty one (58.1%) respondents had faced a life threatening situation in the study contrary to the studies (3,8,11). The reason for differences in the frequency/ total number of emergency could be explained by having several persons reporting the same event occurring in the dental school more than once.

There has not been any published data regarding emergency drugs and equipments recommendations against which we can compare our results. The guidelines differ in recommended drug and equipments, but when a direct comparison is made between commonly recommended emergency drugs conclusions can be drawn. We found that respondents in our study had a good knowledge in identifying the four common drugs like adrenaline, glucose, oxygen and glycerly trinitrate. The knowledge was not at an acceptable level, particularly when discussing drugs like Midazolam, prednisolone and chlorpheniramine maleate and very few respondents recognized these as being essential drugs (8,9) ( Table 1).

Similar results were found when investigating level of knowledge regarding emergency equipments. Single use syringes, oxygen face mask and blood glucose measurement device were recognized to some extent and lowered knowledge was seen regarding equipments like pocket masks, portable suction and self inflating child and adult bag valve mask (9,15) ( Table 2).

The confidence in the use of drugs and equipments mentioned were at a very lower level than the knowledge for all the drugs and equipments mentioned. This suggests that although training is received in the theoretical aspect of emergencies, participants are not particularly confident to treat emergencies and may require further practical training ( Table 1, Table 2, Table 3).

From the responses regarding the number of hours of medical emergencies training undertaken in the undergraduate curriculum, it is evident that there are more definitive guidelines regarding the number of hours of training is recommended. Only 57% of respondents had undergone medical emergency and basic life support (BLS) training for less than 5 hours which was very low. The result may be due to the lack of definitive guidelines about the training with medical emergencies in the dental curriculum (9).

Overall in the study, a large number of graduates stated that they did not know how to proceed in those situations even though they received training in the management of medical emergencies at some time, they expressed the need for further medical emergencies training (3,5,8,9,11,16).

Dentists are members of the medical profession and should be confident in dealing with emergencies which may arise during their work. However our results indicate a worrying picture of the level of competence which the dental graduates have in dealing with emergencies. Both quality and volume of medical emergencies training which dental studies receive should be assessed and improved, to ensure the safety and well being of the public at all times.

## Conclusion

The study showed that syncope is the commonest medical emergency event and the others are hypoglycemic attacks, allergic reactions, epileptic attacks and asthmatic attacks. All in all, dental graduates had a superficial knowledge of medical emergencies, drugs and equipments and they expect this topic should be an integral part of their curriculum. A majority of them perceived the need for further training by means of hands on courses. The study has allowed to find deficiencies in the way the dentists were trained dealing with medical emergencies and identify a need for improvement, be by increasing the volume and quality of training which undergraduates perceive in order to enhance their capability to recognize and manage a medical emergency and to become well qualified practitioners.
